# The role of serum Wisteria floribunda agglutinin-positive Mac-2 binding protein in the assessment of fibrosis in children with chronic hepatitis C

**DOI:** 10.1038/s41598-022-14553-8

**Published:** 2022-07-01

**Authors:** Hitoshi Tajiri, Mitsuyoshi Suzuki, Kazuhiko Bessho, Yoshinori Ito, Jun Murakami, Reiko Hatori, Tomoko Takano, Yoko Miyoshi, Stephen Brooks

**Affiliations:** 1 Department of Pediatrics, Kinki University Faculty of Medicine, 377-2 Ohno-higashi, Osaka-Sayama, Japan; 2 Department of Pediatrics, Juntendo University Faculty of Medicine, Tokyo, Japan; 3 Department of Pediatrics, Graduate School of Medicine, Osaka University, Osaka, Japan; 4 Department of Pediatrics, Nagoya University Graduate School of Medicine, Nagoya, Japan; 5 Division of Pediatrics and Perinatology, Tottori University Faculty of Medicine, Tottori, Japan; 6 Department of Pediatrics, Gunma University Graduate School of Medicine, Maebashi, Japan; 7 Department of Pediatrics, Osaka General Medical Center, Osaka, Japan; 8 Department of Microbiology/Immunology, State University of New York at Buffalo, Buffalo, USA

**Keywords:** Paediatric research, Gastroenterology, Medical research

## Abstract

At present, noninvasive fibrosis markers are not available for the assessment of liver fibrosis in children with chronic hepatitis C. Sixty-three children with chronic hepatitis C were included. Changes in Wisteria floribunda agglutinin-positive Mac-2 binding protein (M2BPGi) levels were evaluated in l3 of 27 treatment-naive patients during the natural course of disease (median 4, range 3–6 years). Changes during treatment were evaluated in 27 of 36 patients for 4 (2–9) years of posttreatment follow-up. There were significant differences in the levels of M2BPGi between control group and HCV F0 group (P = 0.002) and between control group and HCV F1 group (P < 0.001). Receiver operating characteristic curve analysis showed that to discriminate stage F1 fibrosis from F0, the cut-off value was 0.95 for M2BPGi with a sensitivity of 52%, specificity of 90%, and area under the curve of 0.687. A substantial decrease in M2BPGi levels by treatment was shown from 0.98 ± 0.57 at pretreatment to 0.42 ± 0.15 at posttreatment (P < 0.001) in the 27 treated patients. Our study shows new findings that M2BPGi may be useful to predict the presence of a mild degree of fibrosis in children with chronic hepatitis C, and such mild fibrosis may be quickly resolved by treatment.

## Introduction

Recently, various noninvasive methods to evaluate liver fibrosis have been developed, including APRI, FIB-4, biomarkers, and elastography using ultrasonography^1–3^. Although elastography has a high diagnostic ability for liver fibrosis and is widely used, it has several drawbacks, such as the requirement for expensive equipment, limited available facilities, and equipment incompatibility. Blood biomarkers are also widely used to evaluate liver fibrosis and prognosis in patients with chronic hepatitis^4–6^. Recent advances in the noninvasive assessment of liver fibrosis based on serum biomarkers and imaging have been summarized in reviews^4,7^.

Wisteria floribunda agglutinin-positive Mac-2 binding protein (M2BPGi) is a recently recognized liver fibrosis biomarker that was developed as a marker for HCV infection in Japan^8,9^. M2BPGi is an N-glycosylated glycoprotein and is secreted as a ligand of galectin-3 (Mac-2). Many studies have compared the diagnostic accuracy of M2BPGi with that of liver biopsy in identifying liver fibrosis stage in adult patients. In all cases, M2BPGi levels significantly increased as liver fibrosis progressed, which confirmed the utility of M2BPGi for diagnosing liver fibrosis in chronic hepatitis C^10,11^. M2BPGi is now widely used for diagnosing liver fibrosis in chronic liver diseases because it can be easily measured in the serum^9,12,13^. Furthermore, an increase in M2BPGi levels over time has been associated with HCC risk^13–15^. A meta-analysis confirmed the utility of M2BPGi in diagnosing fibrosis and predicting HCC risk^15^.

At present, a liver biopsy is the only reliable method in evaluating liver fibrosis in children^16,17^. No reliable fibrosis markers are available for the assessment of liver fibrosis in children with chronic hepatitis C. In the present study, we measured serum M2BPGi in children with chronic hepatitis C both pre- and posttreatment and found that this noninvasive marker is a useful tool for the evaluation of liver fibrosis in such patients.

## Patients and methods

### Control children for reference levels

We determined reference levels of M2BPGi in 104 children without any kind of liver disease. The control subjects consisted of 68 boys and 36 girls with a median age of 10.5 years (range 4–17). Among those children, platelet counts and serum levels of AST and ALT were assayed and confirmed to be within normal ranges on the same day that serum was collected for an M2BPGi assay. Sera were stored at − 20 °C until assayed. Written informed consent was obtained from a parent and/or legal guardian for study participation of the control child group as well as the chronic hepatitis C child group.

### Reproducibility of M2BPGi measurements

To evaluate the reproducibility of M2BPGi measurements, the marker was assayed twice within a median of three months (range 2–6) in 26 children who visited one of six facilities every three months and whose underlying liver diseases were regarded to be clinically cured or to remain stable in remission. The group consisted of 12 with a prolonged sustained virologic response (SVR) after treatment for chronic hepatitis C, 10 with HBV infection in an inactive carrier stage, two with autoimmune hepatitis in a sustained remission, and two with fatty liver in a stable condition. Some data in the chronic hepatitis C group were utilized for posttreatment measurements as described below.

### Children with chronic hepatitis C

Sixty-three children with chronic hepatitis C who visited one of six participating institutions between January 1996 and November 2012 were included in this study. Thirty-six patients underwent antiviral therapies, and 27 patients were treatment naive. Serial serum stock specimens were asked to send to the Osaka General Medical Center for simultaneous M2PBG measurement for this study. As a result changes in M2BPGi during the natural course of disease were evaluated in l3 of the 27 children with chronic hepatitis C. Changes during treatment were evaluated in 27 of the 36 children who underwent therapy for chronic hepatitis C, all of whom responded to treatment with an SVR. Treatment regimens included combinations of peg-interferon and ribavirin (n = 19), simeprevir plus peg-interferon and ribavirin (n = 3), ledipasvir and sofosbuvir (n = 3), and ribavirin and sofosbuvir (n = 2).

This study was approved by the ethics committee of Osaka General Medical Center. Participation in the study was posted at each participating institution. The study protocol conforms with the ethical guidelines of the Helsinki Declaration.

### Measurement of hepatitis viral markers

Anti-HCV antibodies were determined by ELISA (Abbott, Chicago, IL, USA). HCV RNA was measured by RT-PCR (COBAS AMPLICOR HCV MONITOR assay version 2.0, Roche Diagnostics, Tokyo, Japan). HCV serotyping was determined by detecting antibodies against group-specific recombinant proteins for serotypes l and 2 in the putative HCV NS4 protein region by an enzyme immunoassay. HCV genotyping was performed according to the international classification^18^.

### Measurement of fibrosis markers

M2BPGi measurements were automated using the HISCL-2000 instrument (Sysmex Co., Hyogo, Japan)^6,9^. The measured M2BPGi was indexed with the obtained levels using the following equation: cut-off index (COI) = ([M2BPGi]sample − [M2BPGi]NC)/([M2BPGi]PC − [M2BPGi]NC), where the [M2BPGi] sample is the M2BPGi count of the serum sample, and PC and NC are the positive and negative controls. The positive control was a calibration solution preliminarily standardized to yield a COI value of 1.0.

The AST to platelet ratio index (APRI) was calculated using the following equation: APRI = ((AST/upper limit of normal range of AST) × 100)/platelet count (109/L). FIB-4 was calculated according to the following equation: FIB-4 index = (age [years] × AST[IU/L])/(platelet count [109/L] × (ALT [IU/L])1/2). We calculated the APRI index and FIB-4 index by using the data of age, values of AST and ALT, and platelet count on the same day when serum was collected for the M2BPGi assay.

### Histopathology of the liver

Histopathology of the liver was evaluated using the first liver biopsy specimens from children with chronic hepatitis C who were treatment-naive. Liver biopsy specimens were assessed pathologically based on the New Inuyama Classification of chronic hepatitis^19^. In this classification, chronic hepatic diseases are classified according to the degree of fibrosis (F) as follows: F0 (no fibrosis, the same degree as Ishak stage 0), F1 (fibrosis portal expansion, the same degree as Ishak stage 1–2), F2 (bridging fibrosis, the same degree as Ishak stage 3), F3 (bridging fibrosis with lobular distortion, the same degree as Ishak stage 4), and F4 (cirrhosis, the same degree as Ishak stage 5–6)^19,20^. Additionally, chronic hepatic diseases are classified based on the degree of inflammation and necrosis of hepatocytes. The activities (A) are classified as follows: A0 (no necro-inflammatory reaction), A1 (mild necro-inflammatory reaction), A2 (moderate necro-inflammatory reaction), and A3 (severe necro-inflammatory reaction)^19^.

### Statistical analysis

Data were analyzed with the chi-square test, Student's t-test and Mann–Whitney's U-test. A P value < 0.05 was considered statistically significant. Statistical analyses and receiver operating characteristic curve (ROC) analysis were performed using JMP Pro 12 (SAS Institute Inc., Cary, NC, USA).

## Results

### Subject's characteristics

The baseline characteristics of children with chronic hepatitis C and control children are shown in Table 1. Circulating platelet counts were significantly lower in the HCV group than in the control group, as were the FIB-4 index scores. Both serum AST and ALT levels were significantly higher in the HCV group than in the control group, as were the APRI index scores and M2BPGi levels.

**Table 1:** Baseline characteristics of HCV patients and control children.

	HCV group	Control group	P value
	(n = 63)	(n = 104)	
Sex (M/F)	26/37	44/60	NS
Age (years)	8 (1–17)	10 (4–17)	NS
Platelets (10^9^/L)	27.2 ± 6.2	29.7 ± 6.1	P = 0.013
**Genotype**			
G1	27	/	NA
G2	36	/	NA
HCV-RNA (log IU/ml)	6.0 ± 2.1	/	NA
AST (IU/L)	33.5 ± 16.2	26.9 ± 5.4	P < 0.001
ALT (IU/L)	33.6 ± 35.2	15.3 ± 5.8	P < 0.001
**Fibrosis markers**			
APRI	0.43 ± 0.24	0.31 ± 0.09	P < 0.001
FIB-4	0.19 ± 0.08	0.25 ± 0.09	P < 0.001
M2BPGi	0.95 ± 0.55	0.57 ± 0.19	P < 0.001
**Pathology of the liver**			
Grade (A0/A1/A2/A3)	3/27/3/0	/	NA
Stage (F0/F1/F2/F3/F4)	9/22/2/0/0	/	NA

*NS* not significant, *NA* not applicable.

Thirty-three children with chronic hepatitis C underwent a liver biopsy in this study. No active necroinflammation was observed in three (9%) patients, while mild (A1) and moderate activities (A2) were observed in 27 (82%) and three (9%) patients, respectively. No severe activity (A3) was observed. In terms of liver fibrosis, F0 was noted in nine (27%) patients, and mild (F1) and moderate degrees of liver fibrosis (F2) were observed in 22 (67%) and two (6%) patients, respectively. Advanced liver fibrosis (F3 or F4) was not found among the present children (Table 1).

### Reference values in control children

**Figure 1:**
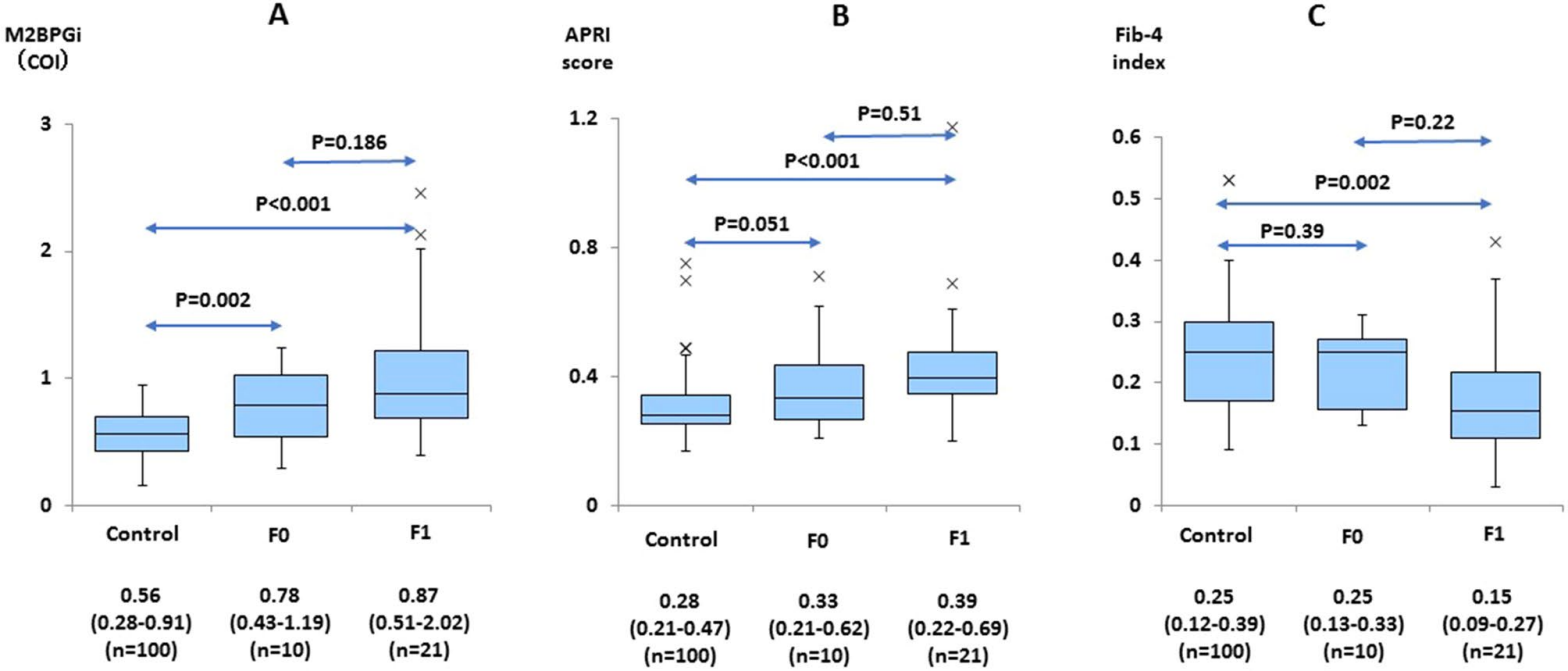
M2BPGi, APRI and FIB-4 levels were expressed by using box-and-whisker plots for control children and for two groups of patients with chronic hepatitis C (F0 and F1). Median value with 95% confidence interval were described for the control, F0 and F1 groups.

M2BPGi levels from 100 of the 104 control children were below 1.0. The remaining four children showed a level greater than 1.0 at the first examination. Repeated assays performed a few months later (3–4 months) showed a level less than 1.0 in all four subjects. After excluding those four children, the median value of M2BPGi with 95% confidence interval (95%CI) for 100 control children was 0.56 (0.28–0.91) (Fig. 1A). The median values with 95%CI for APRI and FIB-4 were 0.28 (0.21–0.47) (Fig. 1B) and 0.25 (0.12–0.39) (Fig. 1C), respectively.

### Reproducibility

The levels of M2BPGi in the first and second assays were 0.42 ± 0.15 and 0.47 ± 0.15, respectively. Differences between the two assays were 0.13 ± 0.08 in 26 children whose underlying liver diseases were clinically cured or stable in remission (Supplementary Fig. 1). Accordingly, a change in M2BPGi of greater than 0.30 COI was arbitrarily regarded as significant in this study because the average plus 2.0 × SD; 0.13 + (0.08 × 2.0) = 0.29.

### Baseline levels of serum M2BPGi in children with chronic hepatitis C

There was a marginal but significant correlation between M2BPGi and APRI (r = 0.332, P = 0.007, n = 46) but not between M2BPGi and FIB-4 index (r = 0.143, P = 0.262, n = 46) (data not shown). The baseline levels of serum M2BPGi in HCV-infected children were greater than those in control subjects (0.95 ± 0.55 vs. 0.57 ± 0.19, P < 0.001) (Table 1).

There was a slight but significant difference in the levels of M2BPGi between the control group and the HCV F0 group (P = 0.002, Fig. 1A). The levels of M2BPGi in the HCV F1 group were also significantly higher than those in the control group (P < 0.001) but not those in the HCV F0 group (P = 0.186, Fig. 1). Because only two patients showed F2 fibrosis, their data were not suitable for this analysis and excluded from Fig. 1A–C.

### ROC analysis

**Figure 2:**
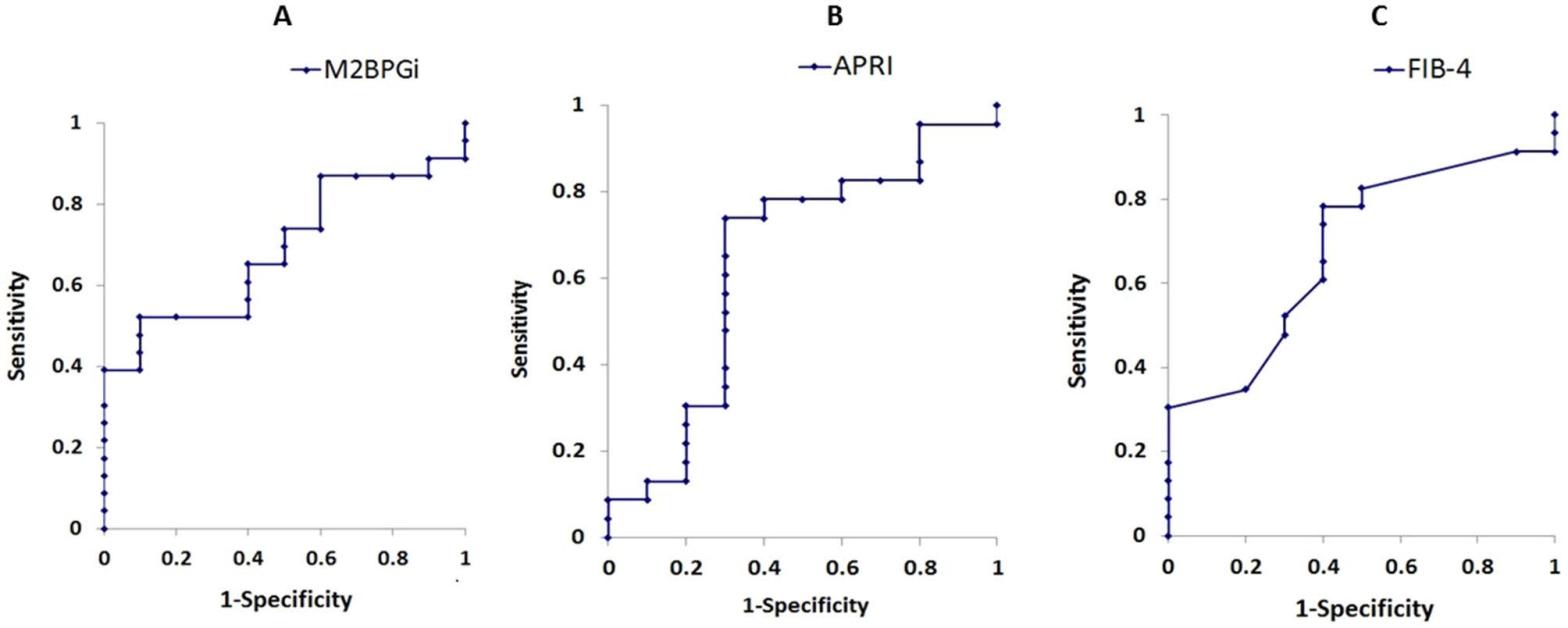
ROC curve analysis of M2BPGI (**A**), APRI (**B**) and FIB-4 (**C**) to discriminate stage F1 fibrosis from F0.

ROC analyses showed that to discriminate stage F1 fibrosis from F0, the cut-off value was 0.95 for M2BPGi, with a sensitivity of 52%, specificity of 90%, and area under the curve (AUROC) of 0.687 (Fig. 2A). The cut-off value was 0.37 for APRI, with a sensitivity of 73%, specificity of 70%, and AUROC of 0.639 (Fig. 2B). The cut-off value was 0.22 for FIB-4 with a sensitivity of 78%, specificity of 60%, and AUROC of 0.680 (Fig. 2C).

### Changes in serum M2BPGi levels in children with chronic hepatitis C during the natural course of disease

Changes in M2BPGi levels during the natural course of disease were evaluated in l3 children with chronic hepatitis C. The median duration from the first assay to the second assay was four years (range 3–6) (Supplementary Fig. 2A). There was no significant change in M2BPGi levels from 0.66 ± 0.33 at the first assay to 0.89 ± 0.42 at the second assay (P = 0.149, Supplementary Fig. 2B). Baseline data of these 13 patients were shown in Supplementary Table 1. The 13 patients were divided into two group by a median value of change in M2BPGi, 0.07 COI:group A with an increase in M2BPGi with a median of 0.79 (range 0.26–0.79), and group B with no apparent increase in M2BPGi with a median of 0.04 (range − 0.33–0.07) (Supplementary Table 2). Group A were all female (P = 0.021) and younger than those in group B (P = 0.127). There were no significant changes regarding other baseline factors including M2BPGi, APRI and FIB-4.

### Changes in serum M2BPGi levels in children with chronic hepatitis C during treatment

**Figure 3:**
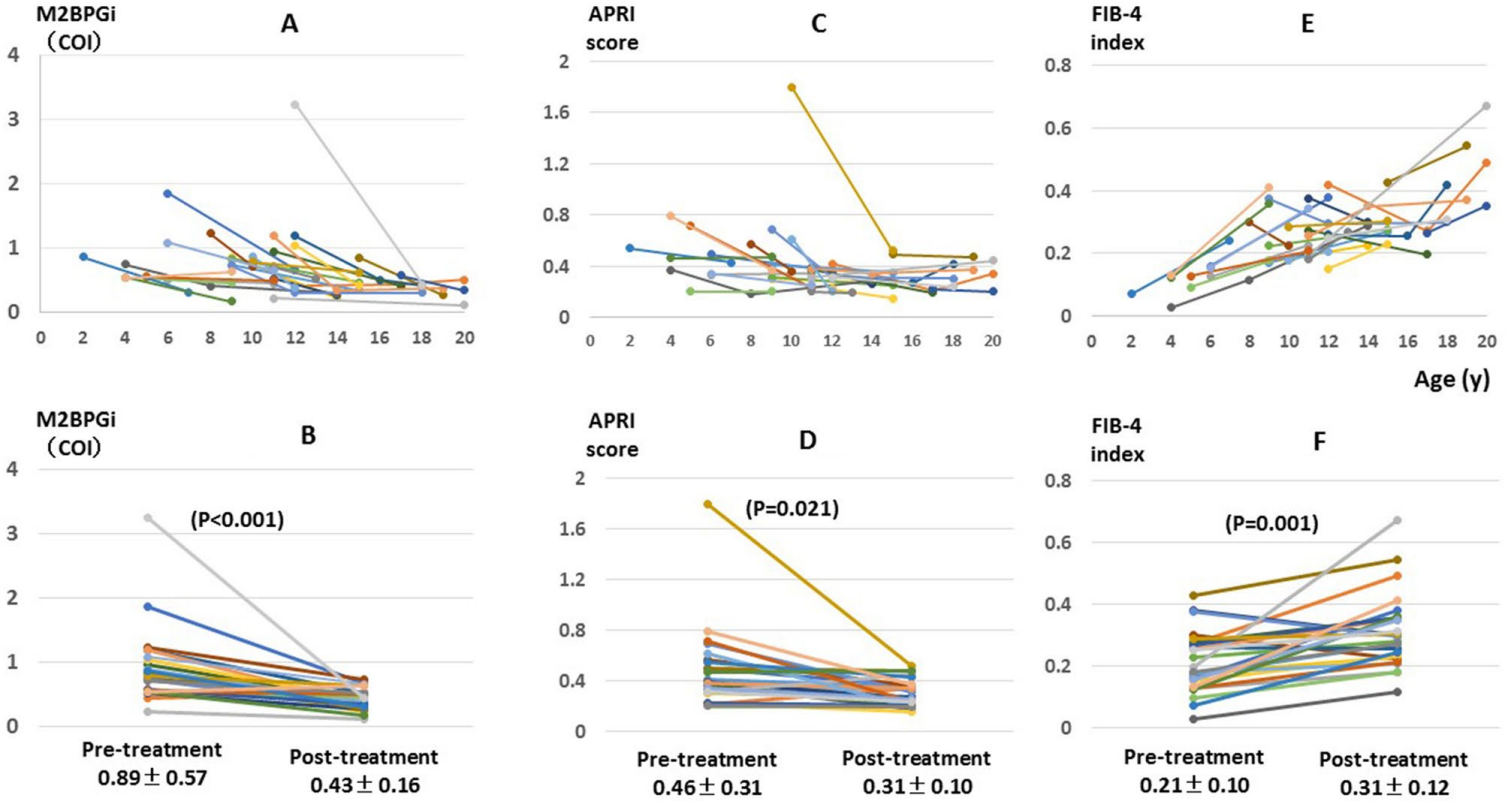
Change in M2BPGi, APRI and FIB-4 levels by treatment. In **A**,**C**,**E** dots indicating M2BPGi levels were plotted at age in years when measurement of M2BPGi was done. M2BPGi dots at pretreatment and at posttreatment are connected for each child in all six Figures.

Changes in M2BPGi levels during treatment were evaluated in 27 patients, all of whom showed an SVR to treatment. The median duration of the follow-up after the end of treatment was 4 years (range 2–9) (Fig. 3A). There was a substantial decrease in M2BPGi levels from 0.89 ± 0.57 pretreatment to 0.43 ± 0.16 posttreatment (P < 0.001, Fig. 3B). Seventeen of the 27 showed a significant change of greater than 0.3 The COI decreased from 1.09 ± 0.61 pretreatment to 0.42 ± 0.16 posttreatment (P < 0.001, data not shown). Regarding APRI there was a significant decrease in APRI scores from 0.46 ± 0.31 to 0.30 ± 0.10 (P = 0.021, Fig. 3C,D). Regarding FIB-4 there was a significant increase in FIB-4 indices from 0.21 ± 0.10 to 0.31 ± 0.12 (P = 0.001, Fig. 3E,F). The changes between pretreatment and post-treatment levels were apparently larger in M2BPGi (P < 0.001) than in APRI and in FIB-4 (P = 0.007 and P = 0.003), respectively (Fig. 3B,D,F).

## Discussion

Previous studies on serum biomarkers, including APRI, FIB-4, and FibroTest, have shown that they are useful for selecting pediatric patients with liver cirrhosis who should undergo a liver biopsy^21–24^. However, chronic hepatitis C generally progresses slowly during childhood, with no cases showing liver cirrhosis in our subjects, consistent with reports in previous studies^25,26^. Whether chronic hepatitis C can lead to a substantial degree of liver fibrosis may be more crucial for a pediatric population. In this regard, the present study has suggested that M2BPGi, one of the noninvasive serum markers, may be useful to predict the presence of a mild degree of fibrosis in children with chronic hepatitis C, and such fibrosis may be quickly resolved by antiviral therapies.

First, we examined the fundamental characteristics of M2BPGi, including reference levels in control children and reproducibility among various types of pediatric chronic liver diseases. Most of the control children showed levels below 1.0 COI which is regarded as a cut-off point for adults. Four of our control subjects showed M2BPGi levels over 1.0 COI and physical examinations performed at that time revealed no symptoms or signs of liver dysfunction, infection, or inflammation. Moreover, repeated assays performed 3–4 months apart in those subjects showed levels below 1.0 COI. The reason for the apparently transient rise in M2BPGi levels is not known at present. However, the marker should be examined repeatedly for reliable evaluation of liver fibrosis.

This study has also shown that M2BPGi may be more useful for predicting the presence of a mild degree of fibrosis (F1) than the fibrosis markers or tests that were already reported. In fact, serum M2BPGi levels from HCV-infected children with F0 or F1 liver fibrosis showed higher levels than the reference levels in control children with no liver disease. The reason for the small elevation in M2BPGi in the F0 group might indicate the presence of minute fibrosis due to a sustained necroinflammatory reaction that is generated by chronic HCV infection. In addition, a better performance of M2BPGi than APRI was suggested by ROC analysis.

This is the first study that performed longitudinal evaluations of M2BPGi along with the natural course of disease and response to treatment in children with chronic hepatitis C. The levels of M2BPGi were found to be elevated during the natural course of disease in some of the reviewed children with chronic hepatitis C. Those with an increase in M2BPGi levels were all female and tended to be younger than those with no apparent increase. Further studies are needed to elucidate the role of gender or age in the production of M2BPGi in children with chronic hepatitis C.

Furthermore, M2BPGi levels were significantly decreased after treatment in children who underwent antiviral treatment, including combination therapy with peg-interferon and ribavirin and direct-acting antiviral agents. In contrast to the significant decrease in M2BPG, FIB-4 was increased by treatment. We speculate that in the children who underwent anti-viral therapies ages were naturally increased, platelet counts were stable and both AST and ALT levels were decreased during treatment. Taken together, contribution of age was apparently large enough to overwhelm those of AST or ALT on calculating FIB-4 index in our patients with treatment and as a result FIB-4 index increased after treatment. Such reverse change in FIB-4 was never reported, and it need further studies how to utilize FIB-4 index in pediatric population with a mild degree of liver fibrosis such as F1.

A literature survey has shown that there have been no reports regarding the role of M2BPGi in children with chronic hepatitis C. There have been some reports on the evaluation of hepatic fibrosis markers associated with chronic hepatitis C: hyaluronic acid, type 4 collagen, APRI, and FIB-4^21–23^. Among them, APRI and FIB-4 are regarded as relatively reliable indices of fibrosis. Our study indicates that M2BPGi showed a better performance than APRI in children with chronic hepatitis C and that FIB-4 might not be reliable in the pediatric group with a mild degree of liver fibrosis.

A limitation of this study is that no HCV-infected patients with advanced liver fibrosis (F3 or F4) were present among our subjects because chronic hepatitis C generally progresses slowly during childhood^25,26^. Another limitation is that this study was not a prospective study, and examination of M2BPGi was not performed in some patients during the observation period of the natural course of disease or in some patients during treatment.

In conclusion, our study shows that M2BPGi may be useful in evaluating the early generation of liver fibrosis in children with chronic hepatitis C as well as in following its resolution after successful treatment. Our study suggests that persistent HCV infection may produce a mild degree of fibrosis during childhood and that such an early pathological change may resolve after antiviral treatment.

## Supplementary Information

Supplementary Information 1.

Supplementary Information 2.

Supplementary Information 3.

Supplementary Information 4.

## Data Availability

The datasets generated during and/or analysed during the current study are available from the corresponding author on reasonable request.

## Abbreviations

HCV: Hepatitis C virus
anti-HCV: Anti-HCV antibody
SVR: Sustained virologic response
IFN: Interferon
RBV: Ribavirin
CHC: Chronic hepatitis C
SD: Standard deviation
DAAs: Direct-acting antiviral agents
