# Supplementary figures and images for: The role of serum Wisteria floribunda agglutinin-positive Mac-2 binding protein in the assessment of fibrosis in children with chronic hepatitis C
Source: Sci Rep. 2022 Jul 1;12:11205. doi: 10.1038/s41598-022-14553-8 (PMC9249794; doi:10.1038/s41598-022-14553-8)

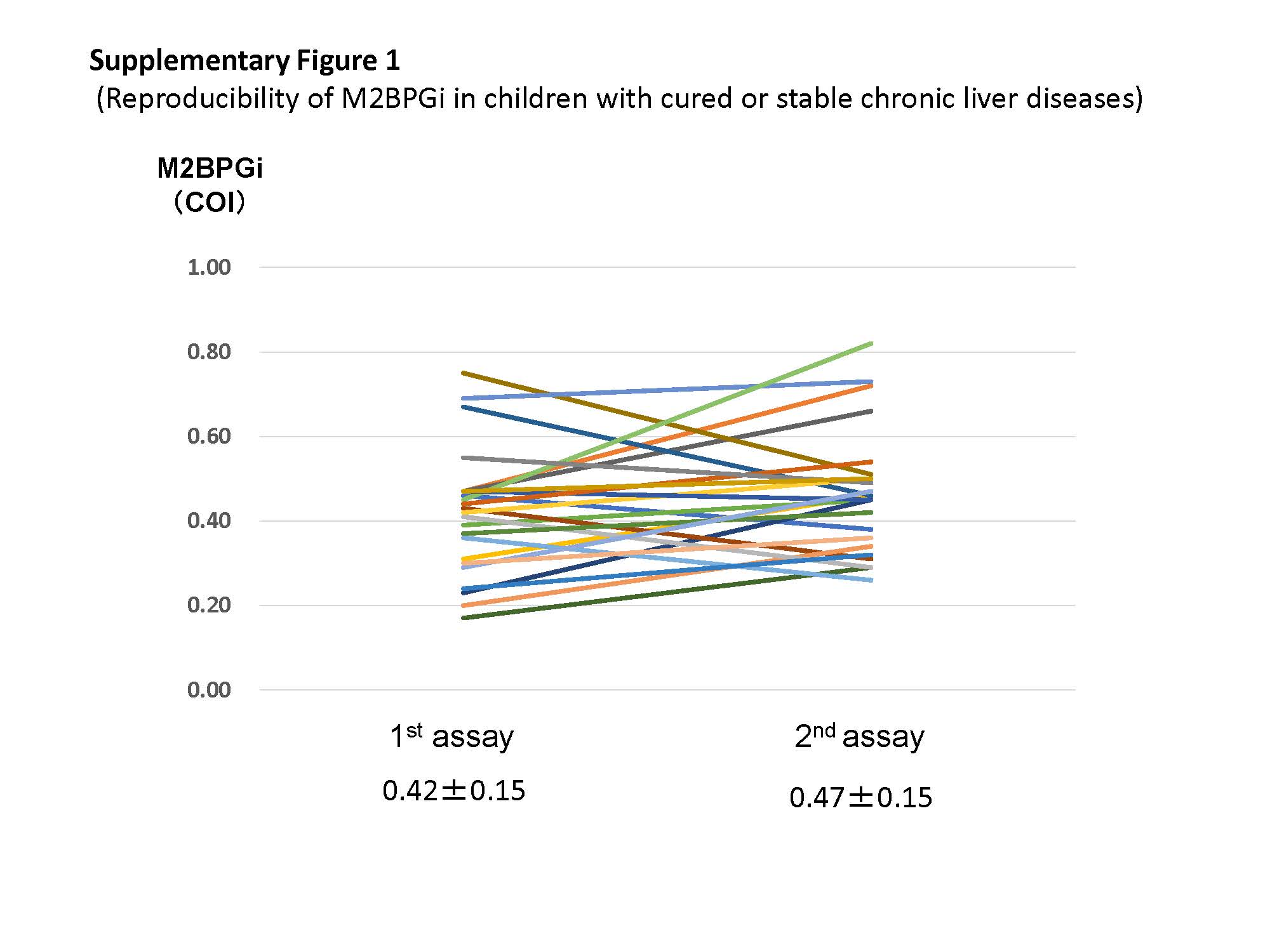

Supplement: Supplementary file 1 — Supplementary Information 1. [file 41598_2022_14553_MOESM1_ESM.jpg]

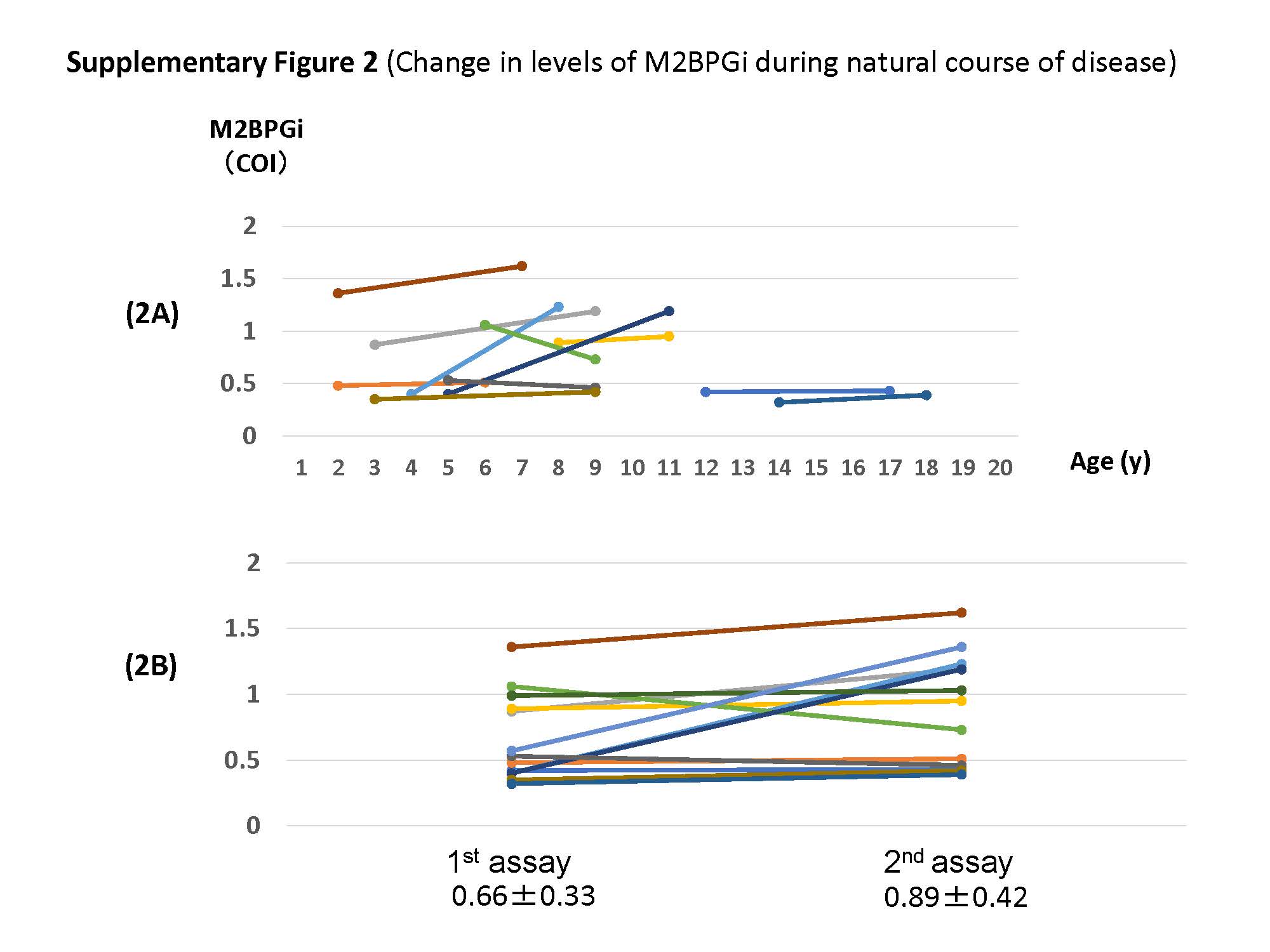

Supplement: Supplementary file 2 — Supplementary Information 2. [file 41598_2022_14553_MOESM2_ESM.jpg]

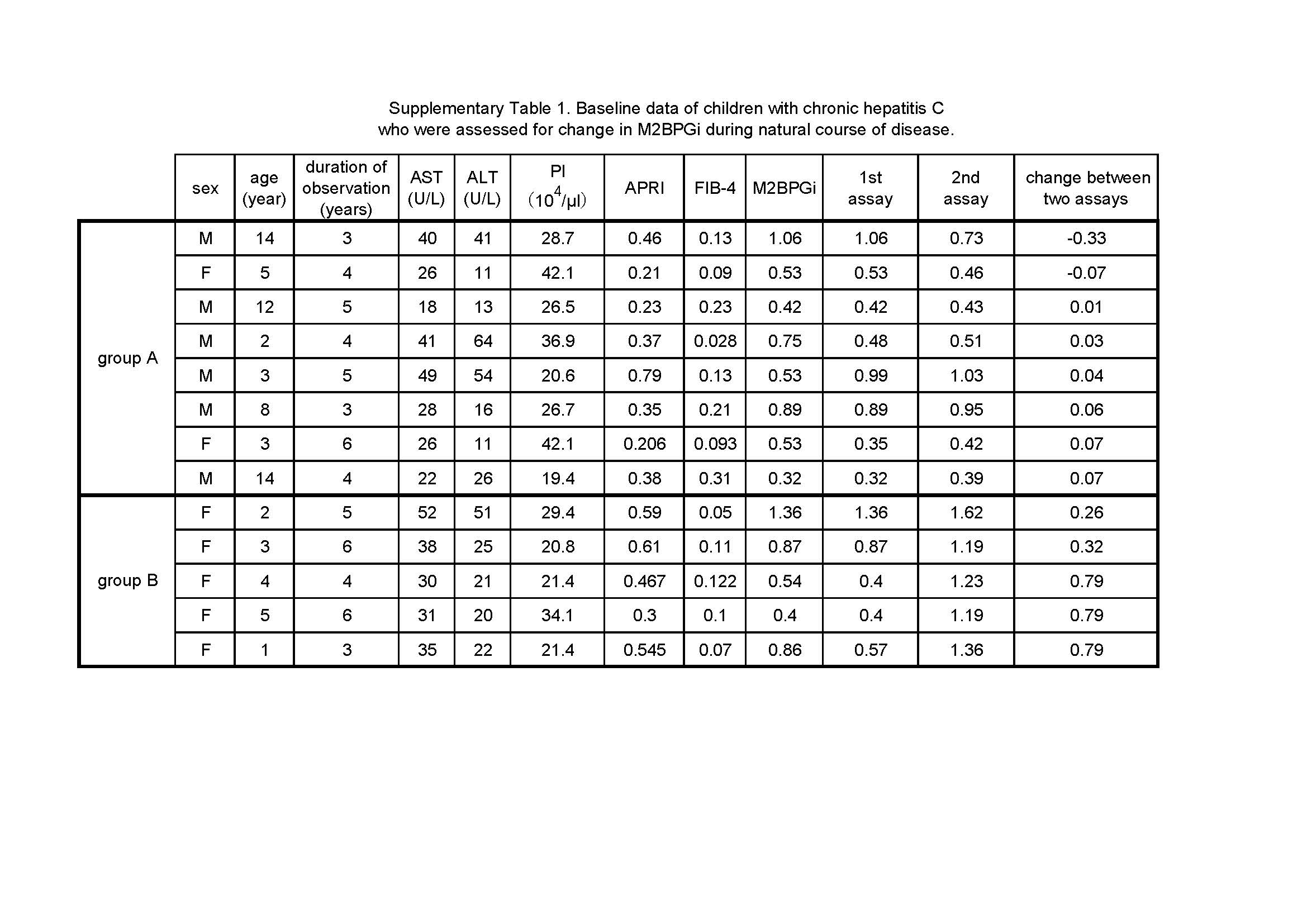

Supplement: Supplementary file 3 — Supplementary Information 3. [file 41598_2022_14553_MOESM3_ESM.jpg]

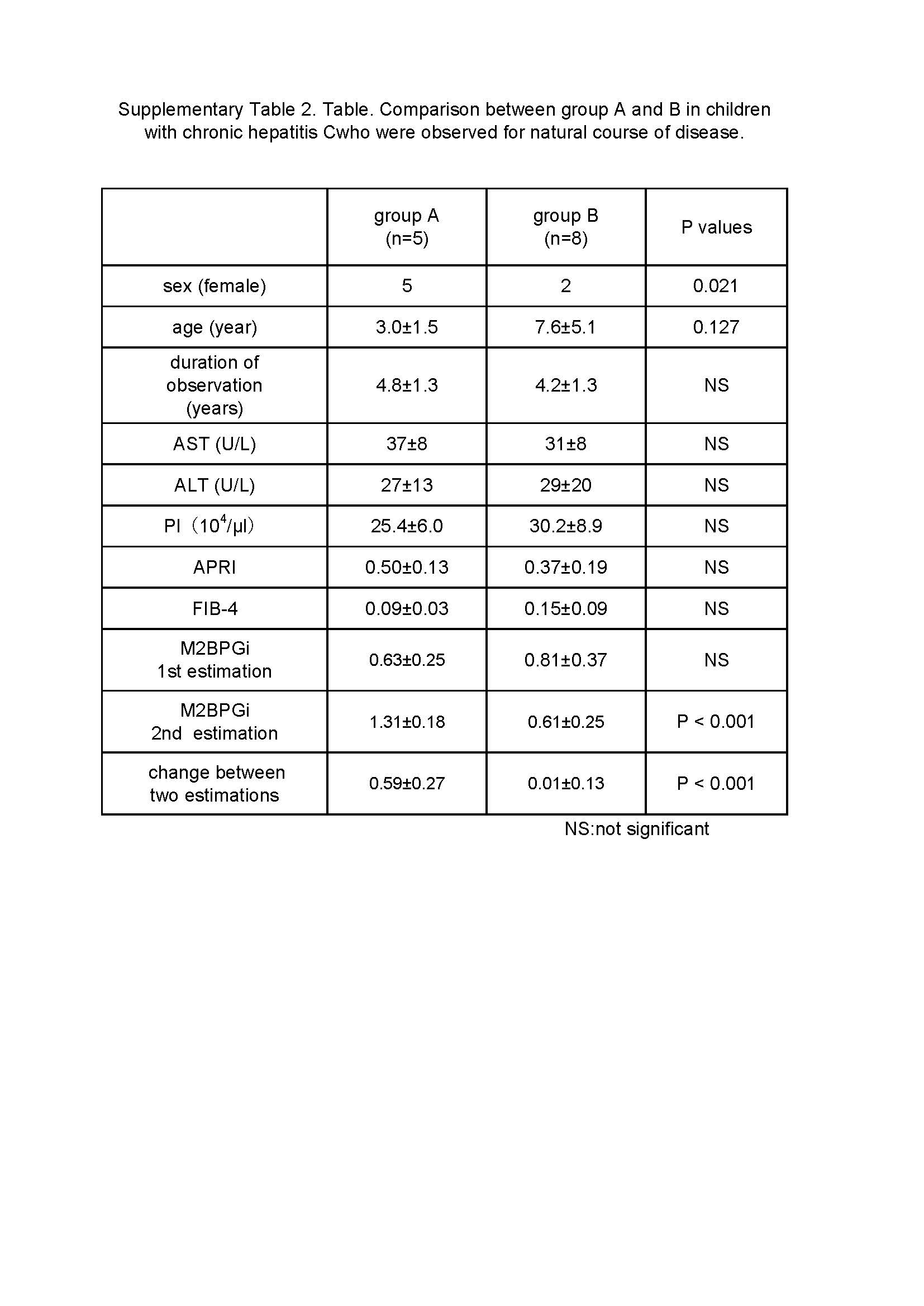

Supplement: Supplementary file 4 — Supplementary Information 4. [file 41598_2022_14553_MOESM4_ESM.jpg]
